# Supplementary material for: A Likelihood Approach for Real-Time Calibration of Stochastic Compartmental Epidemic Models
Source: PLoS Comput Biol. 2017 Jan 17;13(1):e1005257. doi: 10.1371/journal.pcbi.1005257 (PMC5240920; doi:10.1371/journal.pcbi.1005257)
Supplement: S1 File — (TAR.GZ) [file pcbi.1005257.s014.tar.gz › HSPH_Online-SI-Revision/EnKF07/n100-extreme/n100-extreme_PF_EnKF-summery.pdf]

peak -8	R0	$1/\theta_2$	inf	inflog	newly	newlylog	Reff	AttackRate
mean	31.5	58.8	42 078.6	1.6	30 949.2	1.6	67.8	85.3
median	29.4	59.2	5390.	1.6	8837.7	1.7	75.5	83.3
Quantile .1	17.6	37.6	176.7	0.2	358.1	0.4	43.	49.5
Quantile .9	47.5	76.1	98 463.8	2.8	107 892.	2.7	86.7	113.6
45	20	20						
length	50							

peak -4	R0	$1/\theta_2$	inf	inflog	newly	newlylog	Reff	AttackRate
mean	29.9	55.2	345.2	0.7	260.5	0.6	75.8	105.7
median	27.5	54.8	88.8	0.6	108.	0.6	76.1	99.6
Quantile .1	17.8	41.	69.8	0.4	90.5	0.4	68.9	78.3
Quantile .9	46.3	71.	687.7	1.1	676.8	0.9	84.	129.2
47	20	5						
length	50							

peak	R0	$1/\theta_2$	inf	inflog	newly	newlylog	Reff	AttackRate
mean	30.2	51.	86.5	1.	88.6	0.8	50.8	110.2
median	29.6	49.9	88.3	1.	90.2	0.8	49.5	107.2
Quantile .1	12.	36.6	73.4	0.5	77.2	0.5	37.3	84.5
Quantile .9	50.3	68.1	95.2	1.4	96.4	1.2	64.9	140.5
47	26	0						
length	50							

peak +4	R0	$1/\theta_2$	inf	inflog	newly	newlylog	Reff	AttackRate
mean	30.6	52.8	155.7	0.5	253.9	0.5	101.7	116.3
median	29.2	44.8	92.7	0.5	169.1	0.5	93.1	115.
Quantile .1	13.2	21.2	74.2	0.3	87.6	0.3	48.5	80.
Quantile .9	50.6	82.3	203.1	0.8	507.5	0.7	163.8	144.5
46	20	0						
length	50							

peak +8	R0	$1/\theta_2$	inf	inflog	newly	newlylog	Reff	AttackRate
mean	32.5	61.1	1700.	0.7	2139.8	0.8	119.9	110.5
median	28.1	62.7	299.1	0.5	893.5	0.8	115.5	106.1
Quantile .1	15.	30.2	86.8	0.3	253.7	0.5	52.8	84.
Quantile .9	54.9	80.3	3316.3	1.3	4021.8	1.4	188.4	145.6
47	25	0						
length	50							
